# Targeting the Hedgehog Pathway in Rhabdomyosarcoma

**DOI:** 10.3390/cancers15030727

**Published:** 2023-01-24

**Authors:** Patricia Zarzosa, Lia Garcia-Gilabert, Raquel Hladun, Gabriela Guillén, Gabriel Gallo-Oller, Guillem Pons, Julia Sansa-Girona, Miguel F. Segura, Josep Sánchez de Toledo, Lucas Moreno, Soledad Gallego, Josep Roma

**Affiliations:** 1 Childhood Cancer and Blood Disorders, Vall d’Hebron Research Institute (VHIR), Hospital Universitari Vall d’Hebron, Universitat Autònoma de Barcelona, 08035 Barcelona, Spain; 2 Pediatric Oncology and Hematology Department, Hospital Universitari Vall d’Hebron, Universitat Autònoma de Barcelona, 08035 Barcelona, Spain; 3 Pediatric Surgery Department, Hospital Universitari Vall d’Hebron, Universitat Autònoma de Barcelona, 08035 Barcelona, Spain

**Keywords:** cancer, paediatric cancer, soft tissue sarcomas, STS, embryonic pathways, Hh pathway, SMO, Sonic, Indian, Desert, PTCH, SUFU, CDO, BOC, GAS1

## Abstract

**Simple Summary:**

In the first sections of this review, we provide a comprehensive description of the Hedgehog signalling pathway in mammals and the main general models of pathway activation. Subsequently, the review focuses on the oncogenic role played by this pathway in rhabdomyosarcoma and the inhibitors developed to date, as well as the clinical trials available in sarcomas. Finally, we provide a discussion and critical review of the results obtained in the clinical setting and their strong dependency on the type of tumour. In some cases, strong discrepancies between encouraging preclinical data and clinical trial results are clearly evident.

**Abstract:**

Aberrant activation of the Hedgehog (Hh) signalling pathway is known to play an oncogenic role in a wide range of cancers; in the particular case of rhabdomyosarcoma, this pathway has been demonstrated to be an important player for both oncogenesis and cancer progression. In this review, after a brief description of the pathway and the characteristics of its molecular components, we describe, in detail, the main activation mechanisms that have been found in cancer, including ligand-dependent, ligand-independent and non-canonical activation. In this context, the most studied inhibitors, i.e., SMO inhibitors, have shown encouraging results for the treatment of basal cell carcinoma and medulloblastoma, both tumour types often associated with mutations that lead to the activation of the pathway. Conversely, SMO inhibitors have not fulfilled expectations in tumours—among them sarcomas—mostly associated with ligand-dependent Hh pathway activation. Despite the controversy existing regarding the results obtained with SMO inhibitors in these types of tumours, several compounds have been (or are currently being) evaluated in sarcoma patients. Finally, we discuss some of the reasons that could explain why, in some cases, encouraging preclinical data turned into disappointing results in the clinical setting.

## 1. Introduction

Childhood cancers differ from adult malignancies owing to their different aetiology, biology, response to treatment, and outcome. However, despite the small number of cases of childhood cancers compared with the adult population, the understanding of the molecular biology of paediatric tumours has improved considerably in recent years. The pathways often known as *‘embryonic pathways’* (basically Notch, Hedgehog, and Wnt) are appealing candidates as targets for interfering with the mechanisms that drive the oncogenic phenotype of tumour cells. Our understanding of the roles played by these pathways in paediatric tumours is progressing; however, it is far from that of better-known adult malignancies. Nevertheless, recent findings supported these pathways and some of their components as very promising and interesting putative therapeutic targets. 

Rhabdomyosarcoma (RMS) is the commonest type of soft tissue sarcoma in children and adolescents and represents 4–5% of all childhood malignancies. Regarding histopathologic criteria, RMS can be divided into two main subtypes: alveolar and embryonal (ARMS and ERMS, respectively). Molecularly, RMS can be divided into fusion-positive and fusion-negative tumours (in reference of chromosomal translocations affecting mainly *FOXO1* and *PAX3* or *PAX7* genes), a stratification criterion that has been recently demonstrated to be a powerful predictor of prognosis [1,2], since both subtypes differ considerably in their clinical behaviour. The first-line treatment for RMS may include surgery, radiotherapy and chemotherapy, with the most common protocols including Vincristine, Actinomycin D and Cyclophosphamide (VAC) or Ifosfamide, Vincristine and Actinomycin D (IVA) [3].

The clinical and molecular characteristics of RMS tumours, highlighting the cell morphology—similar to that of rhabdomyoblasts—the tumour location—generally in striated muscle, and the expression of various myogenic factors (among others PAX3/7, myogenin and MyoD), have favoured the association of RMS development with a disruption in the proliferation and differentiation of myogenic progenitors [4]. Muscle development begins during embryonic gastrulation and is regulated by the cyclical expression of the aforementioned embryonic pathways and some lineage-specific transcription factors such as PAX3 and PAX7 [5,6], all of them of great relevance for RMS tumours. Moreover, translocation and overexpression of PAX3 and PAX7 genes correlate clinically with poor prognosis, thereby suggesting their significance in RMS progression [7,8]. Additionally, recent studies have described the possibility of originating RMS tumours from non-myogenic cells of adipose and endothelial origin by modulating the embryonic Hedgehog (Hh) pathway, suggesting a pivotal role for this pathway in the genesis of RMS [9,10]. The Hh pathway plays a major role during embryogenesis, where it profoundly influences the fate of a wide range of cells and lineages. Notably, the Hh pathway controls several critical aspects of the myogenic program, the balance of which is also essential during carcinogenesis. It maintains the survival of the most primitive cells of the dermomyotome [11], initiates differentiation by inducing the expression of the myogenic regulatory factors Myf5 [12] and MyoD [13], regulates cell migration of distal limb muscle cells [11], participates in growth of skeletal muscle [14], and, finally, also plays a key role in cell fate determination during adult tissue repair by promoting myofiber regeneration [15].

The contribution of the Hh pathway to cancer was revealed as particularly important. Furthermore, apart from affecting several physiological processes, anomalous activation of the pathway (by deregulation or mutation of some of its components) is often involved in tumorigenesis. The influence of an embryonic pathway, such as Hedgehog, on an embryonic cancer, such as RMS, may not be a coincidence. Thus, the fact that the abnormal regulation of the pathway is quite general in RMS samples (especially in the embryonal subtype, but also in alveolar), together with the fact that mutation of their components—albeit present in some cases—is rare, points to a bi-directional relationship between activation of the pathway and the embryonic status of the original cells.

## 2. Overview of the Hedgehog Signalling Pathway in Mammals

The first step in canonical Hh pathway signalling is the synthesis and maturation of ligands. After their translation, the Hh proteins enter the secretory pathway and undergo autoproteolytic cleavage and two lipidic modifications—the addition of a cholesterol residue at the carboxyl end and the palmitoylation of the amino-terminal end—which causes the release of a 19 kDa bilipidated peptide [16,17]. These modifications confer high hydrophobicity to the Hh ligands, causing their retention in the membrane [18,19]. Once in the membrane, a protein named Dispatched (DISP) interacts with the cholesterol from the Hh ligands and transfers them to the extracellular protein SCUBE, which subsequently solubilizes and releases the Hh ligands [19,20,21]. 

**Figure 1:**
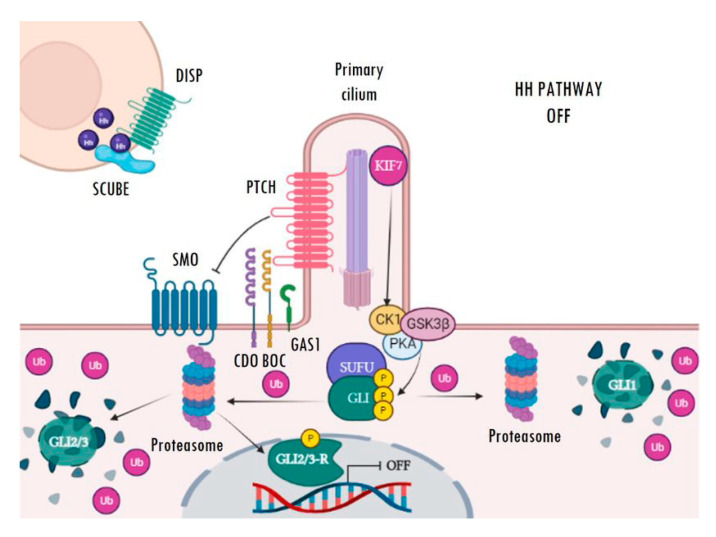
Inactive Hedgehog signalling. In the absence of Hh ligands, PTCH blocks the ciliary localization of SMO. The GLI transcription factors, located at the base of the primary cilium, are inhibited by the SUFU repressor complex. Then, KIF7 mediates GLI phosphorylation via PKA, CK1, and GSK3B kinases. Phosphorylated GLI1 is then ubiquitinated and totally degraded by the proteasome. However, GLI2 and GLI3 can be entirely or partially degraded. Incomplete degradation of GLI2/3 gives rise to the GLI repressor forms (GLI-R), which are translocated to the nucleus and inhibit the transcription of target genes. Image created with BioRender (BioRender.com).

**Figure 2:**
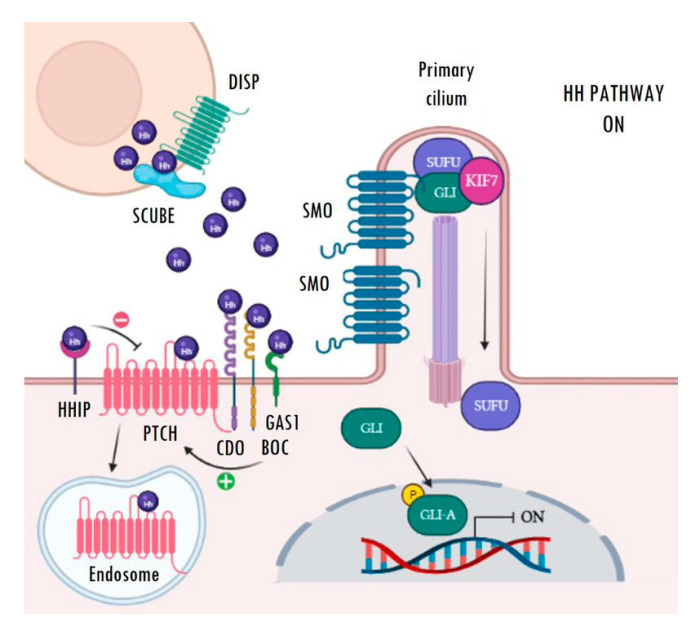
Active Hedgehog signalling. Binding of Hh ligands (purple spheres) to PTCH causes its internalization and subsequent endosomal degradation. Then, SMO moves and accumulates in the primary cilium, where it releases the GLI transcription factors from the repressor complex. Finally, active GLI proteins (GLI-A) translocate to the nucleus, where they are able to activate the transcription of Hh target genes. Image created with BioRender (BioRender.com).

The main receptor of the secreted Hh ligands is the transmembrane protein Patched (PTCH) which, in turn, acts as a constitutive inhibitor of the pathway. In the absence of ligand, PTCH blocks the intermediate modulator Smoothened (SMO) and prevents Hh signalling activation (Figure 1). The binding of Hh ligands to PTCH leads to its subsequent endosomal degradation. The functional inhibition of PTCH allows the activation of SMO and its accumulation in the primary cilium, which facilitates pathway activation resulting in increased transcriptional activity of the pathway’s target genes (Figure 2). The transcriptional activity of Hh targets is mediated by the balance between the repressor (GLI-R) and activator (GLI-A) forms of GLI transcription factors. Thus, in the absence of ligands, GLI and SUFU form a repressor complex that travels to the apical region of the primary cilium. Once there, KIF7 promotes (in a not completely understood mechanism) the sequential hyperphosphorylation of GLI proteins by PKA, CK1, and GSK3B, promoting the proteasomal degradation of its transactivator domain and, in turn, allowing the release of GLI-R, which are rapidly translocated to the nucleus to repress the expression of target genes (Figure 1). Conversely, in the presence of ligands, SMO is translocated to the primary cilium and modulates the role of KIF7; this, instead of promoting GLI proteolysis, causes its dissociation from the repressor complex formed by SUFU and, in turn, allows its release and subsequent nuclear translocation, thereby activating the transcription of Hh target genes (Figure 2) [22,23,24,25,26,27]. Ligand traffic and binding to the PTCH membrane receptor in recipient cells is an incompletely clarified mechanism, with the involvement of several co-adjuvant and little-known membrane proteins, such as CDO, BOC, GAS1, and HHIP. In particular, CDO, BOC, and GAS1 co-receptors interact with the ligand and facilitate its binding to PTCH in an essential manner [16,19,23]. On the contrary, HHIP exerts an antagonist role by competing with PTCH for binding to the ligands.

## 3. General Models of Oncogenic Hh Pathway Activation

### 3.1. Ligand-Independent Hh Activation (Mutational)

**Figure 3:**
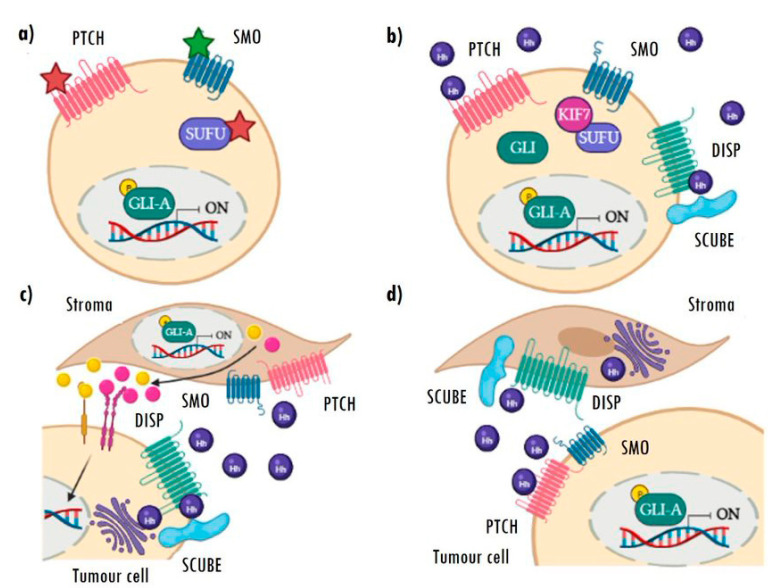
Aberrant mechanisms of Hh pathway activation. (**a**) Type I: ligand-independent constitutive activation caused by inactivating (red star) or activating (green star) mutations in different components of the pathway. (**b**) Type II: autoactivation of cells by high autocrine secretion of ligands. (**c**) Type III: paracrine Hh activation of stromal cells dependent on tumour cell ligand secretion, which, in turn, re-stimulates the tumour via pro-oncogenic Hh target secreted factors. (**d**) Type IIIb: reverse paracrine activation, in which stromal cells secrete Hh ligands and activate signalling in the tumour cells. Image created with BioRender.

Often referred to as type I, ligand-independent Hh activation strongly relies on oncogenic mutations in some of the main actors of the pathway, especially those that are able to induce constitutive activation of the pathway (Figure 3a). This activation increases the transcriptional activity of *GLI* genes among others, thereby increasing the expression of the Hh target genes and promoting tumorigenesis [28]. The most frequent genetic alterations found are mutations leading to PTCH1 inactivation or SMO activation. Nevertheless, loss-of-function mutations in *SUFU* and amplifications and gains of function of the GLI transcription factors, albeit less frequent, have also been described [25,26,27,28,29,30].

The main tumour types associated with this aberrant activation are basal cell carcinoma (BCC) and medulloblastoma (MB). In fact, about 90% of BCCs and more than 30% of sporadic MBs present an aberrant activation of the Hh pathway caused by mutations in *PTCH1* or, to a lesser extent, by mutations in *SMO* [31,32]. Interestingly, an age-dependent molecular heterogeneity in MB has been reported. Thus, the loss of *PTCH1* is the most common in all ages, *SMO* inactivation is particularly frequent in adults and *SUFU* mutations are more common in children [33,34]. The role of SUFU in cancer has become increasingly evident thanks to new publications describing the impact of mutations in this gene. Recently, about 5% of Gorlin Syndrome cases have been associated with mutations in SUFU [28,35]. However, its involvement in the genesis of some tumours (including MB and RMS) appears to be necessary but not sufficient, since heterozygous mice (*SUFU*+/−)—unlike (*PTCH*+/−) mice—require a concomitant loss of *P53* (*P53*−/−) to initiate tumour development [36]. Finally, *GLI1* amplifications, although rare, have been described in various types of sarcomas and childhood brain tumours [37,38] and *GLI2* amplifications have been mainly described in squamous cell carcinoma [39]. However, since these amplicons involve other potential oncogenes, it is difficult to confirm a direct oncogenic role of *GLI* amplifications [40]. Later, genetic studies have permitted the identification of new mutations in the *GLI1* and *GLI3* genes, probably associated with gains of function in breast and pancreatic cancer, as well as polymorphisms in *GLI3* that may predispose to colorectal cancer [41,42,43]. Additionally, the presence of a recently described *GLI1* splicing variant has been associated with increased cell motility and invasiveness in both glioblastoma and breast cancer [44,45].

### 3.2. Ligand-Dependent Hh Activation (Non-Mutational)

Ligand-dependent activation is based on the overexpression of Hh ligands and encompasses types II and III. In type II, once secreted, the ligand is taken up by the tumour cell itself (autocrine activation) or by tumour cells nearby (juxtacrine activation) (Figure 3b). This type of activation is common in breast, prostate, colon, pancreas, ovary, and non-small cell lung cancer, also in hepatocellular carcinomas, melanomas, and gliomas. In addition to the overexpression of Hh ligands, most of these tumours are characterized by presenting an ectopic expression of PTCH1 and GLI [28,29,30,31,46,47,48,49].

In the type III activation model, tumour cells secrete ligands to stimulate pathway activation in stromal cells. This activation in the stroma triggers the expression of Hh target genes and the sending of signals and pro-oncogenic factors to cancer cells, to promote their growth and/or survival (Figure 3c) [28,50]. Cancers with this type of activation show high levels of ligands, with no expression of the pathway’s target genes (*GLI1*, *GLI2*, and *PTCH1*), which are only detectable in stromal cells. Prostate, pancreatic, and colon cancers stand out among the tumours with this type of activation [31,51], which has also been recently described in RMS [52] (for more detail regarding RMS, see Section 4).

There is a new variant of this activation mechanism, known as type IIIb reverse paracrine signalling (Figure 3d). In this case, the stroma secretes Hh ligands and activates signalling in tumour cells. This mechanism has only been described in haematological neoplasms, such as B-cell lymphomas, multiple myelomas, and leukaemias, in which the tumours receive the Hh ligand secreted directly by the stromal cells of the bone marrow or lymph nodes [31,51].

### 3.3. Non-Canonical Hh Activation (Non-Mutational)

The Hh pathway can also be activated or influenced by particular proteins or signalling pathways that are not considered to belong to the Hedgehog pathway. In general, this type of activation is referred to as a non-canonical Hh signalling, and is thought to participate in both transcriptional activation of *GLI* genes and post-translational modifications of GLI proteins. This type of activation has been related to the development of several cancer types with elevated GLI activity. The elements or pathways that can activate GLI proteins mainly include PI3K, KRAS signalling, TGFβ and PKC [53].

The association between the Hh signalling and the PI3K/AKT/mTOR signalling has been found in many tumour entities including oesophageal, ovarian, pancreatic and breast cancers, melanoma, and RMS [53,54]. In the particular case of RMS, the PI3K/AKT/mTOR signalling is often active [55] and may promote GLI1 phosphorylation, which results in its dissociation from the inhibitor SUFU, thus triggering GLI1 activation [56]. Interestingly, the inhibition of GLI1/2 and PI3K/mTOR seems to produce a synergistic effect on apoptosis induction and tumour growth reduction in RMS [54]. Furthermore, in sporadic ERMS, which express Hh target genes apparently without canonical Hh signalling, PI3K/AKT/mTOR inhibitors effectively inhibit Hh target gene expression and cell proliferation, whereas Hh pathway inhibitors alone do not [57]. 

The interaction between Hh and RAS signalling pathways has also been reported in multiple cancer types. For example, oncogenic NRAS and HRAS are able of enhancing the transcriptional activity and nuclear localization of GLI1 in human melanoma cells [58]. Moreover, the oncogenic KRAS is able to increase GLI1 transcriptional activity and protein levels in pancreatic ductal adenocarcinoma [59]. However, although RAS signalling activation is frequent in ERMS tumours, RAS oncogenic mutations (HRAS, KRAS, and NRAS) inhibit GLI1 through the MEK/ERK pathway. [60,61].

Regarding TGF-β signalling, it has been shown that this pathway can induce the expression of GLI1 and GLI2 transcription factors in several cancer cell lines, such as pancreatic ductal adenocarcinoma and breast cancer [62]. Interestingly, combined inhibition of TGF-β and GLI2 reduces self-renewal and survival of cancer stem cells in colorectal cancer [63]. 

Moreover, there is some controversy regarding the regulation of GLI activity by PKCα and PKCδ isoforms, as there are studies supporting both positive and negative regulation of GLI1. Thus, constitutive activation of PKCα decreases GLI1 transcriptional activation, while that of PKCδ increases GLI1 transcriptional activity in HEK-293T cells [53]. Furthermore, the atypical protein kinase C ι/λ (aPKC-ι/λ) has been identified also as a regulator of GLI in mammals. In addition, it has been demonstrated that targeting aPKC-ι/λ suppresses Hh pathway signalling and proliferation of BCC cell lines resistant to the SMO inhibitor Vismodegib [64].

Finally, in addition to the interactions with the better-known pathways described above, many other tumour-specific GLI dependencies have been described. For example, the EWS/FLI translocation driver of Ewing’s sarcoma directly transactivates de GLI1 promoter [65]. Additionally, there are dichotomous modulators such as DYRK1A, which depending on tumour type, can stimulate the Hh pathway by promoting the nuclear translocation of GLI1 or induce its degradation by acting negatively on the cytoskeleton [66]. Another field with much to explore is epigenetic regulation. In this context, the relationship between the tumour suppressor SNF5 (*SMARCB1*) and GLI1 in malignant rhabdoid tumours is particularly suggestive, as about 25% of RMS tumours share the SNF5 mutational inactivation characteristic of rhabdoid tumours [67,68].

## 4. Oncogenic Role of the Hedgehog Pathway in RMS

The involvement of the Hh pathway in the genesis of RMS was first described in the patched knockout mouse by Hahn et al. in 1998, who reported that mice with heterozygous inactivation of PTCH1 had an increased incidence of an embryonal subtype of RMS (ERMS) [29]. Currently, consistent activation of the pathway is well established and commonly accepted in RMS after several works focused on this issue [69,70,71]. Even though the expression of Hh components is also prominent in the alveolar subtype (ARMS) [52], a higher degree of Hh activation in ERMS (and translocation-negative ARMS) has been reported. Furthermore, pathway hyperactivation has been correlated with a worse prognosis [71,72]. Additionally, GLI1 upregulation has also been recently reported to correlate with treatment resistance in RMS and Ewing’s sarcoma (ES), thereby suggesting that GLI1 targeting may benefit patients with RMS and ES by reducing multidrug resistance [73]. Despite the efforts dedicated to this pathway in RMS, the activation mechanism remained elusive, probably caused by the fact that the expression of the most commonly studied ligand of the pathway, the Sonic Hedgehog protein (SHh) is very often negligible in RMS tumours (more than 75% of samples showed no detectable SHh expression) and the prominent expression of the two alternative ligands (Indian (IHh) and Desert (DHh) Hedgehog) was almost always left aside in previous cancer studies. In 2017, a strong expression of these two ligands in almost all RMS patients and cell lines was demonstrated, thereby pointing to a ligand-dependent model as the most plausible mechanism for eliciting pathway activation in the majority of RMS tumours [52]. 

Moreover, a minor but non-negligible percentage of patients bear genetic alterations that can trigger pathway activation in a ligand-independent manner. However, despite all authors agreeing that is not found in the majority of tumours, some controversy exists regarding the importance of this mutation-driven constitutive activation. On the one hand, some works reported that neither *PTCH* mutations nor activating *SMO* mutations appeared to be implicated in pathway activation [70,74]; on the other hand, other authors reported losses in the *PTCH* region 9q22 in one third of ERMS, and loss of *SUFU* has also been reported in 18% of ERMS [75,76]. In a recent work from the Children’s Oncology Group (COG) in highly-differentiated fusion negative tumours, four out of 22 patients harboured mutations in the Hh pathway (three in *PTCH1*, one in *SUFU*) [77]. Additionally, genomic amplification of chromosomal region 12q13-15 containing the *GLI1* gene was identified in a very small subset of ARMS tumours [70,75]. Despite the discrepancies found, activator mutations can only account for pathway activation in relatively small subsets of patients and, therefore, the majority of cases should be considered ligand-dependent activation. 

## 5. Hedgehog Inhibitors and Clinical Trials

### 5.1. SMO Inhibitors

The extensive involvement of the Hh pathway in the oncogenesis and progression of several cancers, including RMS, suggested a marked potential for pathway inhibitors as a putative molecularly-targeted anticancer therapy [78]. These expectations have materialised in the clinical development of several Hh pathway inhibitors, with some having been (or currently being) evaluated in sarcoma patients (Table 1). Most of these compounds target the Hh transmembrane modulator, SMO [79]. In fact, the only Hh pathway inhibitors clinically approved by the Food and Drug Administration (FDA) and the European Medicines Agency (EMA) are SMO inhibitors: Vismodegib (GDC-0449, Erivedge^®^, Genentech, San Francisco, CA, USA) and Sonidegib (LDE225, Odomzo^®^, Sun Pharmaceutical Industries, Mumbai, India), for the treatment of adult BCC [80,81] and Glasdegib (PF-04449913, Daurismo^®^, Pfizer, New York, USA), employed in the treatment of newly-diagnosed acute myeloid leukaemia (AML) [82]. 

SMO inhibitors have shown encouraging results in the treatment of BCC and MB [83,84,85,86,87], both tumours associated with type I Hh activation (mutation-driven). However, SMO inhibitors have not met expectations in tumours associated with ligand-dependent Hh pathway activation, such as sarcomas, colon, ovary, and pancreas [88,89,90]. In fact, much controversy exists about the results observed with SMO inhibitors in tumours other than BCC and MB. In particular, in vitro and in vivo RMS models have shown that SMO inhibitors are not always effective and may even promote tumour progression in some cases [52]. Unfortunately, there are very few clinical trials in RMS and other sarcomas aimed at clarifying whether the preclinical results previously obtained are reproducible in the clinical setting. In the particular case of Vismodegib, only one phase II clinical trial has studied its effect as monotherapy in patients with sarcoma (chondrosarcoma). In this study, after six months of treatment, 25.6% of patients achieved clinical benefit. This was a positive result; however, the objective of the study was to achieve a clinical benefit rate of 40%, thereby reducing the interest in Vismodegib as monotherapy for chondrosarcoma [91,92]. Moreover, Vismodegib was also studied in combination with a Gamma-Secretase (Notch Signalling Pathway) Inhibitor (RO4929097) in a phase Ib/II trial in adult RMS patients and other types of adult sarcomas. However, the development of this Notch inhibitor was discontinued and the phase II trial closed prematurely, despite results demonstrating that the combination therapy was safe. Although the study was not completed, the results analysed showed that Vismodegib did not significantly improve the clinical efficacy of RO4929097 [93,94]. Finally, Sonidegib was evaluated in a phase I trial (which included RMS patients among other recurrent Hh-dependent tumours, including MB). The results demonstrated that children with advanced solid tumours presented good tolerance to Sonidegib. Nevertheless, anti-tumour activity was only observed in the SHh subgroup MB and not in the other tumour types, which were mainly associated with non-mutational Hh pathway activation [83,95]. 

Other compounds developed later, also demonstrated effectivity in preclinical models but their studies in clinical trials have been quite limited. Thus, despite promising preclinical results, TAK-44 was suspended in 2013 due to project prioritization, BMS-833923 (XL139) was withdrawn from the company pipeline in 2014 without disclosing the reason, and the CUR61414 clinical trial was halted due to unsatisfactory results [96]. Finally, another compound named NVP-LEQ506 was moved into phase I trials, as a backup for Sonidegib [97]. 

The possibility of using the novel antifungal agent Posaconazole was recently pointed out; it can downregulate some targets of the Hh pathway (SMO, GLI1, c-MYC, CDK4, and CDK6). This work in vitro and in a murine model provided a theoretical basis that may have important clinical implications in developing Posaconazole as a promising agent against ERMS by targeting the Hedgehog pathway [98].

**Table 1:** Clinical trials with Hh pathway inhibitors in sarcomas.

Study	Clinical Trials. gov Identifier	Inhibitor Name	Activity	Tumour Type	Phase	Outcome
Vismodegib in Treating Patients with Advanced Chondrosarcomas	NCT01267955	Vismodegib (GDC-0449)	SMO inhibitor	Chondrosarcoma	Phase II	Clinical benefit was achieved after 6 months in 25.6% of patients [91]
Vismodegib and Gamma-Secretase/Notch Signalling Pathway Inhibitor RO4929097 in Treating Patients with Advanced or Metastatic Sarcoma	NCT01154452	Vismodegib (GDC-0449)	SMO inhibitor	Adult rhabdomyosarcoma and other advanced/metastatic sarcomas	Phase I Phase II	The combination therapy was safe but Vismodegib did not significantly improve the clinical efficacy of RO4929097 [93]
A Phase I Dose Finding and Safety Study of Oral LDE225 in Children and a Phase II Portion to Assess Preliminary Efficacy in Recurrent or Refractory MB	NCT01125800	Sonidegib (LDE225)	SMO inhibitor	Rhabdomyosarcoma and other paediatric tumours potentially dependent on the Hh pathway	Phase I Phase II	Only the SHh subgroup of medulloblastoma patients, as defined by a five-gene signature RT-PCR assay, responded [95]
A Safety and Efficacy Study of Patients with Metastatic or Locally Advanced (Unresectable) Chondrosarcoma	NCT01310816	Patidegib/Saridegib (IPI-926)	SMO inhibitor	Chondrosarcoma	Phase II	Ended prematurely. On 14 June 2012, a planned futility analysis of data from the study concluded that treatment with IPI-926 was similar to placebo and, therefore, the trial would not meet its primary endpoint
A Study of LY2940680 in Paediatric Medulloblastoma or Rhabdomyosarcoma	NCT01697514	Taladegib (LY2940680)	SMO inhibitor	Medulloblastoma and rhabdomyosarcoma	Phase I	Withdrawn (Trial stopped early for poor accrual)
Arsenic Trioxide in Treating Patients with Advanced Neuroblastoma or Other Childhood Solid Tumours	NCT00024258	Arsenic trioxide	GLI inhibitor	Sarcoma and other paediatric tumours	Phase II	The disease progressed in 72.7% and stabilized in 22.7% of the patients
Arsenic Trioxide Plus Radiation Therapy in Treating Patients with Newly Diagnosed Malignant Glioma	NCT00045565	Arsenic trioxide	GLI inhibitor	Gliosarcoma and other malignant glioma	Phase I	No results posted
Study of Genistein in Paediatric Oncology Patients (UVA-Gen001) (UVA-Gen001)	NCT02624388	Genistein	GLI inhibitor	Sarcoma and other paediatric tumours	Phase II	The therapy is safe and well tolerated

### 5.2. GLI Inhibitors

GLI inhibitors were highlighted as an interesting alternative to SMO inhibitors [99], as they could overcome some of their limitations [100,101,102]. However, most GLI inhibitors are still in preclinical stages, and the only two GLI inhibitors that have been clinically tested were natural molecules, not particularly target-specific. On the one hand, Arsenic trioxide (ATO) was approved by the FDA and the EMA for the treatment of adult acute promyelocytic leukaemia (APL) [103]. Despite its action mechanism not being fully known, it was recently proposed as a GLI inhibitor (among many other targets) [104,105,106]. There was one phase II clinical trial that studied ATO’s effectiveness in children with sarcoma and other childhood solid tumours. In this trial, the only information available was that the disease progressed in 72.7% and stabilized in 22.7% of patients [107,108]. On the other hand, Genistein, the other GLI clinically tested inhibitor, is a natural phytoestrogen with a wide variety of pharmacological properties; it is being extensively studied for its potential anti-tumour effects, affecting different pathways, among which, GLI1 signalling regulation [109,110]. The only existing clinical trial in sarcomas started analysing the effect of Genistein in ES, RMS, non-RMS Soft Tissue Sarcomas, and other paediatric tumours, but was halted prematurely and did not continue because of poor enrolment [111]. Considering the difficulty in inhibiting other transcription factors, the development of specific GLI inhibitors could be a challenge in the future. 

## 6. Hh Inhibitors in RMS: From Encouraging Preclinical Data to Disappointing Clinical Results

Hedgehog inhibitors are thought to be a potential therapeutic alternative in about one third of human cancers (those with the pathway active), and, very often, preclinical data appeared to also corroborate their potential to treat them; however, clinical studies dramatically lowered expectations. In fact, the only approved indications for SMO inhibitors are BCC, MB, and AML, where Hh pathway inhibitors showed clear clinical responses. It may be too simplistic, but as a general principle, it could be stated that SMO inhibitors work acceptably well in solid tumours when they harbour pathway activating mutations, but have not yet been shown to be sufficiently effective in tumours in which the pathway is activated in a ligand-dependent manner or by non-canonical activation. The case of RMS, a type of tumour that does not stand out for harbouring mutations in Hh pathway genes does not constitute an exception to this general rule. Thus, despite evidence rendering the relationship between the Hedgehog pathway and RMS clearly manifest [29,52,69,70,71,112], SMO inhibitors are still a long way from their clinical application in this particular type of sarcoma. 

There are many possible explanations for the clinical failure of SMO inhibitors outside mutation-driven cancers. One reason may be the high concentrations used in preclinical experiments. For instance, even when using in vitro concentrations up to 300-fold higher than the levels required to block SMO in MB, the study in RMS of Hahn et al. concluded that the downregulation of GLI1 was very modest (or, in some cases, GLI1 expression was upregulated). Moreover, in the same study, the effects seen on cell proliferation did not correlate with the degree of GLI1 downregulation [113] and were only observed at concentrations several hundred-fold higher than those required to achieve target inhibition, strongly suggesting remarkable off-target effects. These conclusions from this interesting preclinical work and some others appeared to seriously compromise the potential clinical applicability of these compounds in RMS, since the high doses required are unlikely to be achieved in patients and, if reached, they may likely be accompanied by off-target toxicities. Thus, these studies did not support the use of SMO inhibitors for RMS treatment. In this sense, in a recent review, Dr Tom Curran revealed numerous inconsistencies regarding experimental designs, blaming not only the high complexity of the Hh pathway, but also the existence of an unconscious bias due to the strong expectation regarding the impact of Hh inhibitors on human cancer [114]. The high oncogenic involvement of the Hh pathway has led to the development of inhibitors, without having a complete understanding of the mechanisms that lead to the activation of the pathway, especially in non-mutation-driven cancers, such as RMS. Therefore, it is important to carry out an exhaustive molecular study of the Hh pathway in tumours that do not respond to SMO inhibitors, but that show a strong dependency on Hh ligands, in order to find new molecular targets and an effective way to target them. Particularly for ligand-dependent cancers, the complete characterization of the mechanism of ligand/receptor binding, including the role of the understudied pathway co-activators (CDO, BOC, and GAS1), may be fundamental to develop new inhibitors in the upcoming years, since there are no currently available pharmacological possibilities to inhibit the pathway at this upstream level.

Beyond the use of SMO inhibitors, GLI inhibitors (such as GANT61), may have potential in tumours that express high levels of these proteins and may have a broader range of indications since they may function regardless of the presence of Hh-activating mutations. GANT61 has shown to inhibit GLI activity in preclinical models that also leads to tumour growth impairment [115]. GANT61 is a compound reported in 2007 from a GLI-luciferase drug screen that effectively reduced GLI1/2 DNA-binding [116]. However, GANT61 and other agents able to bind GLI proteins have not reached the clinical setting to date, with no trial registered despite more than 15 years have passed since its first description. Other agents, such as ATO and Genistein, have been tested in trials, but these were non-specifically designed to bind GLI proteins and are thought to be far less specific.

Another promising therapeutic strategy is the combination of Hedgehog inhibitors with other agents. Potentially, combined approaches could act on crosstalk compensatory mechanisms with other pathways, which presumably could benefit not only patients with initially resistant tumours but also tumours that develop resistance after receiving targeted Hh monotherapy. However, selecting the ideal combination is highly challenging and requires a deeper understanding of the complex mechanisms involved. Sometimes, although there is biological evidence of interaction, some combinatory strategies are not effective, such as Hedgehog and Notch inhibition in patients with sarcomas [93,94]. In this context, we believe that the aforementioned types of non-canonical activation may provide us with essential guidelines for future research in this promising field. A clear example is the PI3K-AKT pathway, first found upregulated in MB tumours that became resistant to Sonidegib [117], then successfully tested in preclinical models of resistant SMO mutant tumours [118], and now been explored in a combinatory phase Ib Clinical trial of Hh and PI3K inhibitors in advanced solid tumours [119].

In addition, the complexity of the aberrant involvement of Hh in cancer emphasises the need for predictive biomarkers to optimise treatment selection for each patient. This need has already been addressed with SMO inhibitors in MB, where the variable clinical responses correlate to the genomic heterogeneity of the SHH subgroup [87,120,121]. Thus, a report of two Vismodegib phase II studies confirmed the expected exclusive effectiveness of Vismodegib treatment in patients with genetic alterations in SMO upstream Hh components (loss of *PTCH1*). In contrast, SHH MB patients with molecular aberrations of genes downstream of SMO (*GLI2* and *SUFU*) did not respond to therapy. Notably, based on a strong diffuse P53 immunohistochemistry pattern, the authors suggested a link between *TP53* DNA-binding domain mutations, which would favour gene amplification downstream of SMO, and the consequent lack of efficacy of Vismodegib. Therefore, genome sequencing and copy number analysis to identify Hh pathway mutations and potential cooperating mutations were proposed for future trials [87]. However, these analyses are not suitable for translation to the clinic given the time and quantity of samples required. Therefore, a five-gene Hedgehog signature assayed in formalin-fixed paraffin-embedded samples by RT-PCR was developed as a patient preselection tool for Hedgehog inhibitor therapy in MB [122]. This five-gene Hedgehog signature (up-regulation of *GLI1*, *SHROOM2*, *SPHK1*, and *PDLIM3* and down-regulation of *OTX2*) strongly correlated with response in Sonidegib-treated BCC and MB patients [121,123]. Additionally, Sonidegib and Vismodegib exhibited dose- and exposure-dependent inhibition of *GLI1* in tumour and normal skin biopsies. However, *GLI1* inhibition didn’t correlate with tumour responses, pointing at *GLI1* as a good pharmacodynamic marker for SMO inhibitors but not for tumour response [87,123]. Unfortunately, no predictive biomarkers have been identified for the majority of tumour types, including RMS. The fact that all these findings are relevant exclusively for ligand-independent tumours (BCC and MB), hopes lie in further understanding of the different dependencies of these two ways of aberrant Hedgehog activation. In the case of RMS, its low incidence makes it even more challenging to find and validate potential biomarkers. However, we consider that enrolment of the small subset of ERMS patients with *PTCH1* loss in clinical trials of SMO inhibitors could potentially benefit patients and provide valuable new molecular data.

## 7. Conclusions

Pathological activation of the Hh signalling pathway is known to play an oncogenic role in a wide range of solid tumours. Among them, those that are often associated with oncogenic mutations in several components of the pathway (MB and BCC) clearly stand out. In this particular cancer types, SMO inhibition has demonstrated strong clinical potential and, specifically for these tumours, three SMO inhibitors have already reached the clinical setting, rendering encouraging results. In these cases, the in vitro doses of SMO inhibitors required to inhibit the pathway are relatively low. On the other hand, in the tumour types in which pathway activation strongly relies on the presence of Hh ligands, as in RMS, the results are far less promising. Thus, the in vitro concentrations required to achieve anti-oncogenic effects in cell lines are very high (up to 600 times higher than in MB) and, very often, the specific Hh pathway target downregulation observed (such as in GLI proteins) are very disappointing, thereby suggesting an off-target toxicity that may explain why the positive effects observed in cell lines are not reproducible in the clinical setting. The development of GLI inhibitors is still incipient, with the majority of specific GLI compounds in preclinical stages. Only two naturally occurring compounds, ATO and Genistein, have reached clinical trials; but, in this case, the compounds were not designed to specifically bind GLI proteins, and can downregulate GLI proteins, along with many other targets. Another interesting point is the non-canonical activation, which very often is difficult to be distinguished from the canonical ligand-dependent. The non-canonical activation is thought to have an important role in RMS, since there is solid evidence that several pathways (especially PI3K/AKT/mTOR and RAS/MEK/ERK) can promote GLI protein activation in this particular type of cancer, thereby suggesting the possibility of using combined therapies which putatively may benefit patients with refractory tumours or even tumours that develop resistance after receiving Hh monotherapy. In this sense, the implementation of predictive biomarkers to personalize treatment for each patient would enormously help. This need has already been partially addressed with SMO inhibitors in MB (clinical responses correlate to the genomic heterogeneity within SHH subgroup), but we are still far away from this scenario in the majority of tumours (including RMS), since no predictive Hh-pathway biomarkers have been identified to date. Finally, the less characterised part of the pathway, the binding of ligands to the PTCH receptor and co-receptors CDO, BOC, and GAS1, may harbour strong potential for the development of future therapies, but these upstream elements, located in or related to the plasma membrane, remain still largely unexplored. 

## References

[1] Hibbitts E., Chi Y.Y., Hawkins D.S., Barr F.G., Bradley J.A., Dasgupta R., Meyer W.H., Rodeberg D.A., Rudzinski E.R., Spunt S.L. (2019). Refinement of Risk Stratification for Childhood Rhabdomyosarcoma Using FOXO1 Fusion Status in Addition to Established Clinical Outcome Predictors: A Report from the Children’s Oncology Group. Cancer Med..

[2] Gallego S., Zanetti I., Orbach D., Ranchère D., Shipley J., Zin A., Bergeron C., de Salvo G.L., Chisholm J., Ferrari A. (2018). Fusion Status in Patients with Lymph Node-Positive (N1) Alveolar Rhabdomyosarcoma Is a Powerful Predictor of Prognosis: Experience of the European Paediatric Soft Tissue Sarcoma Study Group (EpSSG). Cancer.

[3] Chen C., Dorado Garcia H., Scheer M., Henssen A.G. (2019). Current and Future Treatment Strategies for Rhabdomyosarcoma. Front. Oncol..

[4] Merlino G., Helman L.J. (1999). Rhabdomyosarcoma—Working out the Pathways. Oncogene.

[5] Chal J., Pourquié O. (2017). Making Muscle: Skeletal Myogenesis in Vivo and in Vitro. Development.

[6] Bryson-Richardson R.J., Currie P.D. (2008). The Genetics of Vertebrate Myogenesis. Nat. Rev. Genet..

[7] Manceau L., Albert J.R., Lollini P.L., Greenberg M.V.C., Gilardi-Hebenstreit P., Ribes V. (2022). Divergent Transcriptional and Transforming Properties of PAX3-FOXO1 and PAX7-FOXO1 Paralogs. PLoS Genet..

[8] Skapek S.X., Anderson J., Barr F.G., Bridge J.A., Gastier-Foster J.M., Parham D.M., Rudzinski E.R., Triche T., Hawkins D.S. (2013). PAX-FOXO1 Fusion Status Drives Unfavorable Outcome for Children with Rhabdomyosarcoma: A Children’s Oncology Group Report. Pediatr. Blood Cancer.

[9] Hatley M.E., Tang W., Garcia M.R., Finkelstein D., Millay D.P., Liu N., Graff J., Galindo R.L., Olson E.N. (2012). A Mouse Model of Rhabdomyosarcoma Originating from the Adipocyte Lineage. Cancer Cell.

[10] Drummond C.J., Hanna J.A., Garcia M.R., Devine D.J., Heyrana A.J., Finkelstein D., Rehg J.E., Hatley M.E. (2018). Hedgehog Pathway Drives Fusion-Negative Rhabdomyosarcoma Initiated From Non-Myogenic Endothelial Progenitors. Cancer Cell.

[11] Hu J.K.H., Mcglinn E., Harfe B.D., Kardon G., Tabin C.J. (2012). Autonomous and Nonautonomous Roles of Hedgehog Signaling in Regulating Limb Muscle Formation. Genes Dev..

[12] Anderson C., Williams V.C., Moyon B., Daubas P., Tajbakhsh S., Buckingham M.E., Shiroishi T., Hughes S.M., Boryck A.G. (2012). Sonic Hedgehog Acts Cell-Autonomously on Muscle Precursor Cells to Generate Limb Muscle Diversity. Genes Dev..

[13] Voronova A., Coyne E., Al Madhoun A., Fair J.V., Bosiljcic N., St-Louis C., Li G., Thurig S., Wallace V.A., Wiper-Bergeron N. (2013). Hedgehog Signaling Regulates MyoD Expression and Activity. J. Biol. Chem..

[14] Bren-Mattison Y., Hausburg M., Olwin B.B. (2011). Growth of Limb Muscle Is Dependent on Skeletal-Derived Indian Hedgehog. Dev. Biol..

[15] Norris A.M., Johnson C.D., Zhou L.Y., Appu A., McKellar D.W., Cosgrove B.D., Kopinke D. (2022). Hedgehog Signaling Acts as Cell Fate Determinant during Adult Tissue Repair. bioRxiv.

[16] Qi X., Li X. (2020). Mechanistic Insights into the Generation and Transduction of Hedgehog Signaling. Trends Biochem. Sci.

[17] Yang L., Xie G., Fan Q., Xie J. (2010). Activation of the Hedgehog-Signaling Pathway in Human Cancer and the Clinical Implications. Oncogene.

[18] Ingham P.W., McMahon A.P. (2001). Hedgehog Signaling in Animal Development: Paradigms and Principles. Genes Dev..

[19] Lee R.T.H., Zhao Z., Ingham P.W. (2016). Hedgehog Signalling. Development.

[20] Chen H., Liu Y., Li X. (2020). Structure of Human Dispatched-1 Provides Insights into Hedgehog Ligand Biogenesis. Life Sci. Alliance.

[21] Tukachinsky H., Kuzmickas R.P., Jao C.Y., Liu J., Salic A. (2012). Dispatched and Scube Mediate the Efficient Secretion of the Cholesterol-Modified Hedgehog Ligand. Cell Rep..

[22] Cheung H.O.L., Zhang X., Ribeiro A., Mo R., Makino S., Puviindran V., Lo Law K.K., Briscoe J., Hui C.C. (2009). The Kinesin Protein Kif7 Is a Critical Regulator of Gli Transcription Factors in Mammalian Hedgehog Signaling. Sci. Signal..

[23] Ryan K.E., Chiang C. (2012). Hedgehog Secretion and Signal Transduction in Vertebrates. J. Biol. Chem..

[24] Bangs F., Anderson K.V. (2017). Primary Cilia and Mammalian Hedgehog Signaling. Cold Spring Harb. Perspect. Biol..

[25] Teglund S., Toftgård R. (2010). Hedgehog beyond Medulloblastoma and Basal Cell Carcinoma. Biochim. Biophys. Acta.

[26] Heretsch P., Tzagkaroulaki L., Giannis A. (2010). Modulators of the Hedgehog Signaling Pathway. Bioorg Med. Chem..

[27] Matise M.P., Wang H. (2011). Sonic Hedgehog Signaling in the Developing CNS. Where It Has Been and Where It Is Going. Curr. Top. Dev. Biol..

[28] Sigafoos A.N., Paradise B.D., Fernandez-Zapico M.E. (2021). Hedgehog/Gli Signaling Pathway: Transduction, Regulation, and Implications for Disease. Cancers.

[29] Hahn H., Wojnowski L., Zimmer A.M., Hall J., Miller G., Zimmer A. (1998). Rhabdomyosarcomas and Radiation Hypersensitivity in a Mouse Model of Gorlin Syndrome. Nat. Med..

[30] Reifenberger J., Wolter M., Knobbe C.B., Köhler B., Schönicke A., Scharwächter C., Kumar K., Blaschke B., Ruzicka T., Reifenberger G. (2005). Somatic Mutations in the PTCH, SMOH, SUFUH and TP53 Genes in Sporadic Basal Cell Carcinomas. Br. J. Dermatol..

[31] Skoda A.M., Simovic D., Karin V., Kardum V., Vranic S., Serman L. (2018). The Role of the Hedgehog Signaling Pathway in Cancer: A Comprehensive Review. Bosn J. Basic. Med. Sci..

[32] Doheny D., Manore S.G., Wong G.L., Lo H.W. (2020). Hedgehog Signaling and Truncated GLI1 in Cancer. Cells.

[33] Northcott P.A., Hielscher T., Dubuc A., MacK S., Shih D., Remke M., Al-Halabi H., Albrecht S., Jabado N., Eberhart C.G. (2011). Pediatric and Adult Sonic Hedgehog Medulloblastomas Are Clinically and Molecularly Distinct. Acta Neuropathol..

[34] Kool M., Jones D.T.W., Jäger N., Northcott P.A., Pugh T.J., Hovestadt V., Piro R.M., Esparza L.A., Markant S.L., Remke M. (2014). Genome Sequencing of SHH Medulloblastoma Predicts Genotype-Related Response to Smoothened Inhibition. Cancer Cell.

[35] Huq A.J., Walsh M., Rajagopalan B., Finlay M., Trainer A.H., Bonnet F., Sevenet N., Winship I.M. (2018). Mutations in SUFU and PTCH1 Genes May Cause Different Cutaneous Cancer Predisposition Syndromes: Similar, but Not the Same. Fam. Cancer.

[36] Lee Y., Kawagoe R., Sasai K., Li Y., Russell H.R., Curran T., McKinnon P.J. (2007). Loss of Suppressor-of-Fused Function Promotes Tumorigenesis. Oncogene.

[37] Roberts W.M., Douglass E.C., Peiper S.C., Houghton P.J., Look A.T. (1989). Amplification of the Gli Gene in Childhood Sarcomas. Cancer Res..

[38] Wasson J.C., Saylors R.L., Zeltzer P., Friedman H.S., Bigner S.H., Burger P.C., Bigner D.D., Look A.T., Douglass E.C., Brodeur G.M. (1990). Oncogene Amplification in Pediatric Brain Tumors. Cancer Res..

[39] Snijders A.M., Schmidt B.L., Fridlyand J., Dekker N., Pinkel D., Jordan R.C.K., Albertson D.G. (2005). Rare Amplicons Implicate Frequent Deregulation of Cell Fate Specification Pathways in Oral Squamous Cell Carcinoma. Oncogene.

[40] Raju G.P., Pham D. (2012). Hedgehog Inhibition as an Anti-Cancer Strategy. Vitam Horm..

[41] Sjöblom T., Jones S., Wood L.D., Parsons D.W., Lin J., Barber T.D., Mandelker D., Leary R.J., Ptak J., Silliman N. (2006). The Consensus Coding Sequences of Human Breast and Colorectal Cancers. Science.

[42] Jones S., Zhang X., Parsons D.W., Lin J.C.H., Leary R.J., Angenendt P., Mankoo P., Carter H., Kamiyama H., Jimeno A. (2008). Core Signaling Pathways in Human Pancreatic Cancers Revealed by Global Genomic Analyses. Science.

[43] Lascorz J., Försti A., Chen B., Buch S., Steinke V., Rahner N., Holinski-Feder E., Morak M., Schackert H.K., Görgens H. (2010). Genome-Wide Association Study for Colorectal Cancer Identifies Risk Polymorphisms in German Familial Cases and Implicates MAPK Signalling Pathways in Disease Susceptibility. Carcinogenesis.

[44] Cao X., Geradts J., Dewhirst M.W., Lo H.W. (2012). Upregulation of VEGF-A and CD24 Gene Expression by the TGLI1 Transcription Factor Contributes to the Aggressive Behavior of Breast Cancer Cells. Oncogene.

[45] Lo H.W., Zhu H., Cao X., Aldrich A., Ali-Osman F. (2009). A Novel Splice Variant of GLI1 That Promotes Glioblastoma Cell Migration and Invasion. Cancer Res..

[46] Kinzler K.W., Ruppert J.M., Bigner S.H., Vogelstein B. (1988). The GLI Gene Is a Member of the Kruppel Family of Zinc Finger Proteins. Nature.

[47] Hahn H., Wicking C., Zaphiropoulos P.G., Gailani M.R., Shanley S., Chidambaram A., Vorechovsky I., Holmberg E., Unden A.B., Gillies S. (1996). Mutations of the Human Homolog of Drosophila Patched in the Nevoid Basal Cell Carcinoma Syndrome. Cell.

[48] Reifenberger J., Wolter M., Weber R.G., Megahed M., Ruzicka T., Lichter P., Reifenberger G. (1998). Missense Mutations in SMOH in Sporadic Basal Cell Carcinomas of the Skin and Primitive Neuroectodermal Tumors of the Central Nervous System. Cancer Res..

[49] Taylor M.D., Liu L., Raffel C., Hui C.C., Mainprize T.G., Zhang X., Agatep R., Chiappa S., Gao L., Lowrance A. (2002). Mutations in SUFU Predispose to Medulloblastoma. Nat. Genet..

[50] Tian H., Callahan C.A., Dupree K.J., Darbonne W.C., Ahn C.P., Scales S.J., De Sauvage F.J. (2009). Hedgehog Signaling Is Restricted to the Stromal Compartment during Pancreatic Carcinogenesis. Proc. Natl. Acad. Sci. USA.

[51] Scales S.J., de Sauvage F.J. (2009). Mechanisms of Hedgehog Pathway Activation in Cancer and Implications for Therapy. Trends Pharmacol. Sci..

[52] Almazán-Moga A., Zarzosa P., Molist C., Velasco P., Pyczek J., Simon-Keller K., Giralt I., Vidal I., Navarro N., Segura M.F. (2017). Ligand-Dependent Hedgehog Pathway Activation in Rhabdomyosarcoma: The Oncogenic Role of the Ligands. Br. J. Cancer.

[53] Pietrobono S., Gagliardi S., Stecca B. (2019). Non-Canonical Hedgehog Signaling Pathway in Cancer: Activation of GLI Transcription Factors beyond Smoothened. Front Genet.

[54] Graab U., Hahn H., Fulda S. (2015). Identification of a Novel Synthetic Lethality of Combined Inhibition of Hedgehog and PI3K Signaling in Rhabdomyosarcoma. Oncotarget.

[55] Petricoin E.F., Espina V., Araujo R.P., Midura B., Yeung C., Wan X., Eichler G.S., Johann D.J., Qualman S., Tsokos M. (2007). Phosphoprotein Pathway Mapping: Akt/Mammalian Target of Rapamycin Activation Is Negatively Associated with Childhood Rhabdomyosarcoma Survival. Cancer Res..

[56] Wang Y., Ding Q., Yen C.J., Xia W., Izzo J.G., Lang J.Y., Li C.W., Hsu J.L., Miller S.A., Wang X. (2012). The Crosstalk of MTOR/S6K1 and Hedgehog Pathways. Cancer Cell.

[57] Geyer N., Ridzewski R., Bauer J., Kuzyakova M., Dittmann K., Dullin C., Rosenberger A., Schildhaus H.U., Uhmann A., Fulda S. (2018). Different Response of Ptch Mutant and Ptch Wildtype Rhabdomyosarcoma Toward SMO and PI3K Inhibitors. Front. Oncol..

[58] Stecca B., Mas C., Clement V., Zbinden M., Correa R., Piguet V., Beermann F., Ruiz I., Altaba A. (2007). Melanomas Require HEDGEHOG-GLI Signaling Regulated by Interactions between GLI1 and the RAS-MEK/AKT Pathways. Proc. Natl. Acad. Sci. USA.

[59] Ji Z., Mei F.C., Xie J., Cheng X. (2007). Oncogenic KRAS Activates Hedgehog Signaling Pathway in Pancreatic Cancer Cells. J. Biol. Chem..

[60] Dehner C.A., Armstrong A.E., Yohe M., Shern J.F., Hirbe A.C. (2021). Genetic Characterization, Current Model Systems and Prognostic Stratification in PAX Fusion-Negative vs. PAX Fusion-Positive Rhabdomyosarcoma. Genes.

[61] Bauer J., Cuvelier N., Ragab N., Simon-Keller K., Nitzki F., Geyer N., Botermann D.S., Elmer D.P., Rosenberger A., Rando T.A. (2021). Context-Dependent Modulation of Aggressiveness of Pediatric Tumors by Individual Oncogenic RAS Isoforms. Oncogene.

[62] Dennler S., André J., Alexaki I., Li A., Magnaldo T., Ten Dijke P., Wang X.J., Verrecchia F., Mauviel A. (2007). Induction of Sonic Hedgehog Mediators by Transforming Growth Factor-Beta: Smad3-Dependent Activation of Gli2 and Gli1 Expression in Vitro and in Vivo. Cancer Res..

[63] Tang Y.A., Chen Y.F., Bao Y., Mahara S., Yatim S.M.J.M., Oguz G., Lee P.L., Feng M., Cai Y., Tan E.Y. (2018). Hypoxic Tumor Microenvironment Activates GLI2 via HIF-1α and TGF-Β2 to Promote Chemoresistance in Colorectal Cancer. Proc. Natl. Acad. Sci. USA.

[64] Atwood S.X., Li M., Lee A., Tang J.Y., Oro A.E. (2013). GLI Activation by Atypical Protein Kinase C ι/λ Regulates the Growth of Basal Cell Carcinomas. Nature.

[65] Zwerner J.P., Joo J., Warner K.L., Christensen L., Hu-Lieskovan S., Triche T.J., May W.A. (2008). The EWS/FLI1 Oncogenic Transcription Factor Deregulates GLI1. Oncogene.

[66] Schneider P., Miguel Bayo-Fina J., Singh R., Kumar Dhanyamraju P., Holz P., Baier A., Fendrich V., Ramaswamy A., Baumeister S., Martinez E.D. (2015). Identification of a Novel Actin-Dependent Signal Transducing Module Allows for the Targeted Degradation of GLI1. Nat. Commun..

[67] Jagani Z., Mora-Blanco E.L., Sansam C.G., McKenna E.S., Wilson B., Chen D., Klekota J., Tamayo P., Nguyen P.T.L., Tolstorukov M. (2010). Loss of the Tumor Suppressor Snf5 Leads to Aberrant Activation of the Hedgehog-Gli Pathway. Nat. Med..

[68] DeCristofaro M.F., Betz B.L., Wang W., Weissman B.E. (1999). Alteration of HSNF5/INI1/BAF47 Detected in Rhabdoid Cell Lines and Primary Rhabdomyosarcomas but Not Wilms’ Tumors. Oncogene.

[69] Zibat A., Missiaglia E., Rosenberger A., Pritchard-Jones K., Shipley J., Hahn H., Fulda S. (2010). Activation of the Hedgehog Pathway Confers a Poor Prognosis in Embryonal and Fusion Gene-Negative Alveolar Rhabdomyosarcoma. Oncogene.

[70] Pressey J.G., Anderson J.R., Crossman D.K., Lynch J.C., Barr F.G. (2011). Hedgehog Pathway Activity in Pediatric Embryonal Rhabdomyosarcoma and Undifferentiated Sarcoma: A Report from the Children’s Oncology Group. Pediatr. Blood Cancer.

[71] Satheesha S., Manzella G., Bovay A., Casanova E.A., Bode P.K., Belle R., Feuchtgruber S., Jaaks P., Dogan N., Koscielniak E. (2016). Targeting Hedgehog Signaling Reduces Self-Renewal in Embryonal Rhabdomyosarcoma. Oncogene.

[72] Manzella G.W., Schäfer B. (2016). Interfering with Hedgehog Pathway: New Avenues for Targeted Therapy in Rhabdomyosarcoma. Curr. Drug Targets.

[73] Yoon J.W., Lamm M., Chandler C., Iannaccone P., Walterhouse D. (2020). Up-Regulation of GLI1 in Vincristine-Resistant Rhabdomyosarcoma and Ewing Sarcoma. BMC Cancer.

[74] Calzada-Wack J., Schnitzbauer U., Walch A., Wurster K.H., Kappler R., Nathrath M., Hahn H. (2002). Analysis of the PTCH Coding Region in Human Rhabdomyosarcoma. Hum. Mutat..

[75] Bridge J.A., Liu J., Qualman S.J., Suijkerbuijk R., Wenger G., Zhang J., Wan X., Baker K.S., Sorensen P., Barr F.G. (2002). Genomic Gains and Losses Are Similar in Genetic and Histologic Subsets of Rhabdomyosarcoma, Whereas Amplification Predominates in Embryonal with Anaplasia and Alveolar Subtypes. Genes Chromosom. Cancer.

[76] Tostar U., Malm C.J., Meis-Kindblom J.M., Kindblom L.G., Toftgård R., Undén A.B. (2006). Deregulation of the Hedgehog Signalling Pathway: A Possible Role for the PTCH and SUFU Genes in Human Rhabdomyoma and Rhabdomyosarcoma Development. J. Pathol..

[77] Teot L.A., Schneider M., Thorner A.R., Tian J., Chi Y.Y., Ducar M., Lin L., Wlodarski M., Grier H.E., Fletcher C.D.M. (2018). Clinical and Mutational Spectrum of Highly Differentiated, Paired Box 3:Forkhead Box Protein O1 Fusion–Negative Rhabdomyosarcoma: A Report from the Children’s Oncology Group. Cancer.

[78] Georg-August-Universität Göttingen. https://ediss.uni-goettingen.de/handle/11858/00-1735-0000-0028-8687-C.

[79] Chahal K.K., Parle M., Abagyan R. (2018). Hedgehog Pathway and Smoothened Inhibitors in Cancer Therapies. Anti. Cancer Drugs.

[80] Axelson M., Liu K., Jiang X., He K., Wang J., Zhao H., Kufrin D., Palmby T., Dong Z., Russell A.M. (2013). Food and Drug Administration Approval: Vismodegib for Recurrent, Locally Advanced, or Metastatic Basal Cell Carcinoma. Clin. Cancer Res..

[81] Burness C.B. (2015). Sonidegib: First Global Approval. Drugs.

[82] Hoy S.M. (2019). Glasdegib: First Global Approval. Drugs.

[83] Kieran M.W., Chisholm J., Casanova M., Brandes A.A., Aerts I., Bouffet E., Bailey S., Leary S., Macdonald T.J., Mechinaud F. (2017). Phase i Study of Oral Sonidegib (LDE225) in Pediatric Brain and Solid Tumors and a Phase II Study in Children and Adults with Relapsed Medulloblastoma. Neuro. Oncol..

[84] Chang A.L.S., Solomon J.A., Hainsworth J.D., Goldberg L., McKenna E., Day B.M., Chen D.M., Weiss G.J. (2014). Expanded Access Study of Patients with Advanced Basal Cell Carcinoma Treated with the Hedgehog Pathway Inhibitor, Vismodegib. J. Am. Acad. Dermatol..

[85] Dummer R., Guminksi A., Gutzmer R., Lear J.T., Lewis K.D., Chang A.L.S., Combemale P., Dirix L., Kaatz M., Kudchadkar R. (2020). Long-Term Efficacy and Safety of Sonidegib in Patients with Advanced Basal Cell Carcinoma: 42-Month Analysis of the Phase II Randomized, Double-Blind BOLT Study. Br J. Dermatol..

[86] Rudin C.M., Hann C.L., Laterra J., Yauch R.L., Callahan C.A., Fu L., Holcomb T., Stinson J., Gould S.E., Coleman B. (2009). Treatment of Medulloblastoma with Hedgehog Pathway Inhibitor GDC-0449. N. Engl. J. Med..

[87] Robinson G.W., Orr B.A., Wu G., Gururangan S., Lin T., Qaddoumi I., Packer R.J., Goldman S., Prados M.D., Desjardins A. (2015). Vismodegib Exerts Targeted Efficacy against Recurrent Sonic Hedgehog—Subgroup Medulloblastoma: Results from Phase II Pediatric Brain Tumor Consortium Studies PBTC-025B and PBTC-032. J. Clin. Oncol..

[88] Berlin J., Bendell J.C., Hart L.L., Firdaus I., Gore I., Hermann R.C., Mulcahy M.F., Zalupski M.M., Mackey H.M., Yauch R.L. (2013). A Randomized Phase II Trial of Vismodegib versus Placebo with FOLFOX or FOLFIRI and Bevacizumab in Patients with Previously Untreated Metastatic Colorectal Cancer. Clin. Cancer Res..

[89] Kaye S.B., Fehrenbacher L., Holloway R., Amit A., Karlan B., Slomovitz B., Sabbatini P., Fu L., Yauch R.L., Chang I. (2012). A Phase II, Randomized, Placebo-Controlled Study of Vismodegib as Maintenance Therapy in Patients with Ovarian Cancer in Second or Third Complete Remission. Clin. Cancer Res..

[90] De Jesus-Acosta A., Sugar E.A., O’Dwyer P.J., Ramanathan R.K., Von Hoff D.D., Rasheed Z., Zheng L., Begum A., Anders R., Maitra A. (2020). Phase 2 Study of Vismodegib, a Hedgehog Inhibitor, Combined with Gemcitabine and Nab-Paclitaxel in Patients with Untreated Metastatic Pancreatic Adenocarcinoma. Br J. Cancer.

[91] ClinicalTrials.gov. https://clinicaltrials.gov/ct2/show/NCT01267955.

[92] Italiano A., Le Cesne A., Bellera C., Piperno-Neumann S., Duffaud F., Penel N., Cassier P., Domont J., Takebe N., Kind M. (2013). GDC-0449 in Patients with Advanced Chondrosarcomas: A French Sarcoma Group/US and French National Cancer Institute Single-Arm Phase Ii Collaborative Study. Ann. Oncol..

[93] ClinicalTrials.gov. https://clinicaltrials.gov/ct2/show/NCT01154452.

[94] Gounder M.M., Rosenbaum E., Wu N., Dickson M.A., Sheikh T.N., D’Angelo S.P., Chi P., Keohan M.L., Erinjeri J.P., Antonescu C.R. (2022). A Phase Ib/II Randomized Study of RO4929097, a Gamma-Secretase or Notch Inhibitor with or without Vismodegib, a Hedgehog Inhibitor, in Advanced Sarcoma. Clin. Cancer Res..

[95] Clinicaltrials.gov. https://clinicaltrials.gov/ct2/show/NCT01125800.

[96] Nguyen N.M., Cho J. (2022). Hedgehog Pathway Inhibitors as Targeted Cancer Therapy and Strategies to Overcome Drug Resistance. Int. J. Mol. Sci..

[97] Xin M., Ji X., De La Cruz L.K., Thareja S., Wang B. (2018). Strategies to Target the Hedgehog Signaling Pathway for Cancer Therapy. Med. Res. Rev..

[98] Sun J., Lin W., Li C., Ueki H., Xue R., Sadahira T., Hu H., Wada K., Li N., Liu C. (2021). Repurposing of Posaconazole as a Hedgehog/SMO Signaling Inhibitor for Embryonal Rhabdomyosarcoma Therapy. Am. J. Cancer Res..

[99] Peer E., Tesanovic S., Aberger F. (2019). Next-Generation Hedgehog/GLI Pathway Inhibitors for Cancer Therapy. Cancers.

[100] Berardozzi S., Bernardi F., Infante P., Ingallina C., Toscano S., De Paolis E., Alfonsi R., Caimano M., Botta B., Mori M. (2018). Synergistic Inhibition of the Hedgehog Pathway by Newly Designed Smo and Gli Antagonists Bearing the Isoflavone Scaffold. Eur. J. Med. Chem..

[101] Severini L.L., Quaglio D., Basili I., Ghirga F., Bufalieri F., Caimano M., Balducci S., Moretti M., Romeo I., Loricchio E. (2019). A Smo/Gli Multitarget Hedgehog Pathway Inhibitor Impairs Tumor Growth. Cancers.

[102] Ng J.M.Y., Curran T. (2011). The Hedgehog’s Tale: Developing Strategies for Targeting Cancer. Nat. Rev. Cancer.

[103] European Medicines Agency. https://www.ema.europa.eu/en/medicines/human/EPAR/arsenic-trioxide-accord.

[104] Pubchem. https://pubchem.ncbi.nlm.nih.gov/compound/14888.

[105] Beauchamp E.M., Ringer L., Bulut G., Sajwan K.P., Hall M.D., Lee Y.C., Peaceman D., Özdemirli M., Rodriguez O., Macdonald T.J. (2011). Arsenic Trioxide Inhibits Human Cancer Cell Growth and Tumor Development in Mice by Blocking Hedgehog/GLI Pathway. J. Clin. Investig..

[106] Kim J., Lee J.J., Kim J., Gardner D., Beachy P.A. (2010). Arsenic Antagonizes the Hedgehog Pathway by Preventing Ciliary Accumulation and Reducing Stability of the Gli2 Transcriptional Effector. Proc. Natl. Acad. Sci. USA.

[107] ClinicalTrials.gov. https://clinicaltrials.gov/ct2/show/NCT00024258.

[108] Clincosm. https://www.clincosm.com/trial/brain-and-cns-tumors-childhood-germ-cell-tumor-extragonadal-new-york.

[109] Zhang L., Li L., Jiao M., Wu D., Wu K., Li X., Zhu G., Yang L., Wang X., Hsieh J.T. (2012). Genistein Inhibits the Stemness Properties of Prostate Cancer Cells through Targeting Hedgehog-Gli1 Pathway. Cancer Lett..

[110] Fan P., Fan S., Wang H., Mao J., Shi Y., Ibrahim M.M., Ma W., Yu X., Hou Z., Wang B. (2013). Genistein Decreases the Breast Cancer Stem-like Cell Population through Hedgehog Pathway. Stem Cell Res. Ther..

[111] Clinicaltrials.gov. https://clinicaltrials.gov/show/NCT02624388.

[112] Gorlin R.J. (1987). Nevoid Basal-Cell Carcinoma Syndrome. Medicine.

[113] Ridzewski R., Rettberg D., Dittmann K., Cuvelier N., Fulda S., Hahn H. (2015). Hedgehog Inhibitors in Rhabdomyosarcoma: A Comparison of Four Compounds and Responsiveness of Four Cell Lines. Front Oncol..

[114] Curran T. (2018). Reproducibility of Academic Preclinical Translational Research: Lessons from the Development of Hedgehog Pathway Inhibitors to Treat Cancer. Open Biol..

[115] Carpenter R.L., Ray H. (2019). Safety and Tolerability of Sonic Hedgehog Pathway Inhibitors in Cancer. Drug. Saf..

[116] Lauth M., Bergström Å., Shimokawa T., Toftgård R. (2007). Inhibition of GLI-Mediated Transcription and Tumor Cell Growth by Small-Molecule Antagonists. Proc. Natl. Acad. Sci. USA.

[117] Buonamici S., Williams J., Morrissey M., Wang A., Guo R., Vattay A., Hsiao K., Yuan J., Green J., Ospina B. (2010). Interfering with Resistance to Smoothened Antagonists by Inhibition of the PI3K Pathway in Medulloblastoma. Sci. Transl. Med..

[118] Dijkgraaf G.J.P., Alicke B., Weinmann L., Januario T., West K., Modrusan Z., Burdick D., Goldsmith R., Robarge K., Sutherlin D. (2011). Small Molecule Inhibition of GDC-0449 Refractory Smoothened Mutants and Downstream Mechanisms of Drug Resistance. Cancer Res..

[119] Clinicaltrials.gov. https://clinicaltrials.gov/ct2/show/NCT01576666.

[120] Yauch R., Januario T., Fu L., Holcomb T., Stinson J., Pujara K., Callahan C., Koeppen H., Reddy J., Von Hoff D. (2009). Abstract A44: Predictive Biomarkers of Efficacy to the Hedgehog Pathway Inhibitor, GDC-0449, in Advanced Basal Cell Carcinoma and Medulloblastoma in Phase I Studies. Mol. Cancer Ther..

[121] Kieran M.W. (2017). Lessons Learned from Diffuse Intrinsic Pontine Glioma: How a Terrible Disease Forced Us to Think Better. Neuro Oncol..

[122] Shou Y., Smithson M. (2015). Evaluating Predictors of Dispersion: A Comparison of Dominance Analysis and Bayesian Model Averaging. Psychometrika.

[123] Rodon J. (2014). An (Only) Partially Established Paradigm of Drug Development of Targeted Therapies. Eur. J. Cancer.

